# Can optical coherence tomography replace fluorescein angiography in detection of ischemic diabetic maculopathy?

**DOI:** 10.1007/s00417-013-2518-x

**Published:** 2013-11-29

**Authors:** Diana Anna Dmuchowska, Pawel Krasnicki, Zofia Mariak

**Affiliations:** Department of Ophthalmology, University Teaching Hospital of Bialystok, 24a M. Sklodowskiej-Curie St., 15-276 Bialystok, Poland

**Keywords:** Ischemic diabetic maculopathy, Retinal thickness, Foveal avascular zone outline, OCT, Fluorescein angiography

## Abstract

**Background:**

We studied the association between (1) the retinal thickness, volume and structure assessed by optical coherence tomography (OCT), and (2) vascular changes around the fovea in FA to define the role of these methods in the detection of ischemic diabetic maculopathy.

**Methods:**

This retrospective observational study included 51 eyes of 51 patients with severe non-proliferative and proliferative diabetic retinopathy, considering that they present advanced ischemic retinal changes. Based on the clinical examination and presence of leakage on FA, patients with clinically significant macular edema were excluded.

On FA, foveal avascular zone (FAZ) corresponds to capillary loss and reflects ischemic processes. Its outline and size were assessed according to the ETDRS standards. Patients were divided in two groups according to the severity of damage of the FAZ outline: ≤ grade 2 (*n* = 28) and ≥ grade 3 (*n* = 23).

As we expected ischemia-related damage, SD-OCT images were evaluated for retinal structure, volume, total thickness with division into the outer and inner retina (to assess the influence of two, non-overlapping blood sources) and ganglion cell layer thickness.

**Results:**

The comparisons revealed that at least currently with described methods:

1. There is no significant association between FAZ outline and retinal volume, total thickness, and thickness of the outer and inner retina and ganglion cell layer.

2. There is no significant association between FAZ outline and following retinal structure characteristics: continuity of the external limiting membrane and inner segment/outer segment junction, and identification of the inner/outer retina boundary.

3. The identification of ganglion cell layer boundaries was significantly more difficult in more advanced FAZ outline grades.

4. FAZ size is not correlated with the retinal thickness and volume.

**Conclusions:**

In patients with severe non-proliferative and proliferative diabetic retinopathy, it is not possible to predict FAZ outline/size based solely on the measurements assessing volume and thickness or retinal structure evaluation on OCT. Consequently, at present OCT cannot replace FA in the detection of ischemic diabetic maculopathy.

## Introduction

Fluorescein angiography (FA) plays an important role in diabetic retinopathy (DR) staging and identification of fluorescein leakage sources for the laser treatment of the macula. At the present time, optical coherence tomography (OCT) has become popular in diagnosing and monitoring of diabetic macular edema and its laser, medical, and surgical treatment. In order to define the role of the two imaging methods in the detection of ischemic diabetic maculopathy without clinically significant macular edema (CSME), we studied the association between (1) the retinal thickness, volume, and structure assessed by OCT, and (2) vascular changes around the fovea in FA.

There are two non-overlapping sources of blood supply to the retina. The outer retina is supplied via diffusion from the choriocapillaris. The inner retina is supplied by the central retinal artery. Macular vasculature consists of two capillary plexuses. Superficial capillary plexus lies in the nerve fiber layer or ganglion cell layer, while deep capillary plexus is located within the inner nuclear layer. There are no superficial capillaries in the foveola itself and in the immediate parafoveal retina, making the fovea dependent on the blood supply from the choriocapillaris. This area represents the foveal avascular zone (FAZ), best assessed in early phase of FA. Pathologic conditions that feature retinal capillary dropout, such as diabetes, may show an enlargement and irregular margins of the FAZ [1]. On the other hand, diabetes also leads to reduced choroidal circulation and thickness [2–4], which might lead to hypoxia of the outer retina. Expecting damage due to ischemia, we evaluated retinal volume, thickness, and structure. In addition to total retinal thickness measurement, we also divided the retinal thickness into the outer and inner retina in order to assess the influence of reduced blood supply from these two sources separately. FAZ is of a larger size in diabetic patients in comparison to controls [5–8] and increases as DR advances [5–8]; however, even in healthy subjects FAZ dimensions range from 0 to 1200 μm [1]. Therefore, our study was mainly focused on FAZ outline, which corresponds to capillary loss and reflects ischemic processes, and seems to be a more reliable parameter with regards to representing the degree of potential blood supply insufficiency to the inner retina as compared to FAZ size. Conrath et al. assessed FAZ outline and size in patients with DR without CSME, and suggested employing FAZ outline as either complementary or in place of FAZ size in relevant studies [8].

Retinal thickness is generally higher in diabetic patients in comparison to controls [9–12], and higher in patients with DR than in those without signs of DR [9–11].

A number of papers on DR compare the retinal thickness and structure on OCT with AF images; however, they mainly deal with diabetic maculopathy with dye leakage [9, 13–15]. In contrast, we focused on diabetic maculopathy without dye leakage, and evaluated the association with FAZ outline. Several recent reports have suggested [16–18] that it is not possible to detect ischemic diabetic maculopathy based on OCT findings. Conversely, Byeon et al., who assessed foveal ganglion cell layer damage in ischemic diabetic maculopathy, postulated that OCT provides objective results and seems to be a good noninvasive substitute for FA [19]. Moreover, Van Dijk et al. proved that the duration of diabetes is significantly correlated with ganglion cell layer (GCL) thickness [20]. As the data from the literature is inconclusive, the present study addressed the question whether it is possible to predict the foveal avascular zone (FAZ) outline and size, and thus detect ischemic maculopathy, based on the retinal thickness measurement and retinal structure evaluation on OCT.

## Materials and methods

This retrospective observational cross-sectional study included 51 eyes of 51 patients. The characteristics are summarized in Table 1. The study was performed according to the Declaration of Helsinki, and was approved by the ethics committee at the Medical University of Bialystok. All patients gave written informed consent prior to all examinations.

**Table 1:** Baseline characteristics of patients enrolled into the study

	All	FAZ outline grade 0, 1, 2	FAZ outline grade 3, 4	*P*
*N*	51	28	23	
Age (years)	55.1 ± 13.8	59.4 ± 10.2	49.7 ± 15.9	0.019
Female/male	30/21	18/10	12/11	0.408
Severe NPDR/PDR	16/35	11/17	5/18	0.232

FAZ = foveal avascular zone; NPDR = non-proliferative diabetic retinopathy; PDR = proliferative diabetic retinopathy

We performed visual acuity testing, tonometry, and slit-lamp biomicroscopy. DR and CSME staging were defined according to the Early Treatment Diabetic Retinopathy Study Research Group (ETDRS) [21–23]. We included patients with severe non-proliferative diabetic retinopathy (NPDR) and proliferative diabetic retinopathy (PDR), as they present advanced ischemic retinal changes. Moreover, this group is often omitted in studies, which tend to focus on earlier stages of DR.

**Fig. 1:**
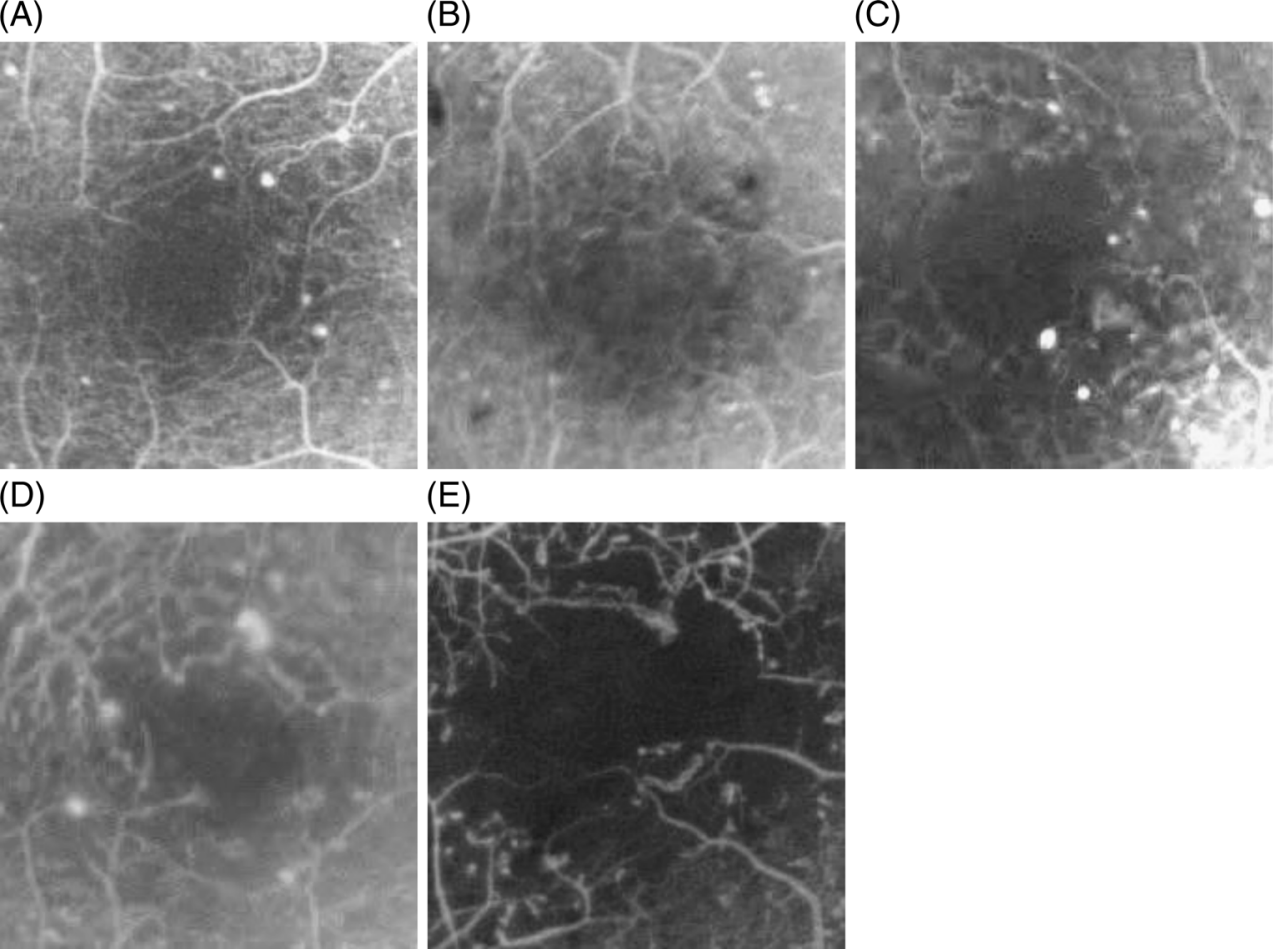
Examples of fluorescein angiograms presenting FAZ outlines of study patients: **a** grade 0 = normal; **b** grade 1 = questionable, outline not smoothly round or oval, but visible irregularities not definitely abnormal; **c** grade 2 = outline definitely destroyed in less than one half the original circumference; **d** grade 3 = outline definitely destroyed for one half or more of the original circumference, but some remnants remain; **e** grade 4 = capillary outline completely destroyed; grade 8 = cannot grade (according to the ETDRS report number 11: “Classification of diabetic retinopathy from fluorescein angiograms” [24]). Angiograms with grade 8 of FAZ outline were excluded from the study

Fluorescein angiography (Kowa Co. Ltd., Tokyo, Japan) was performed according to the standard procedure, and used to assess the severity of DR, characterize the FAZ according to ETDRS standards [24], and exclude CSME defined as dye leakage within 500 μm radius from the fovea or 1,500 μm radius in cases with hard exudates in this area. An early phase frame (after 20–30 seconds) and a late frame (after 3–5 minutes) were selected for further analysis. Severity of perifoveal capillary occlusion was characterized by the outline and size of the FAZ. Examples of FAZ outlines are shown on Fig. 1.

**Fig. 2:**
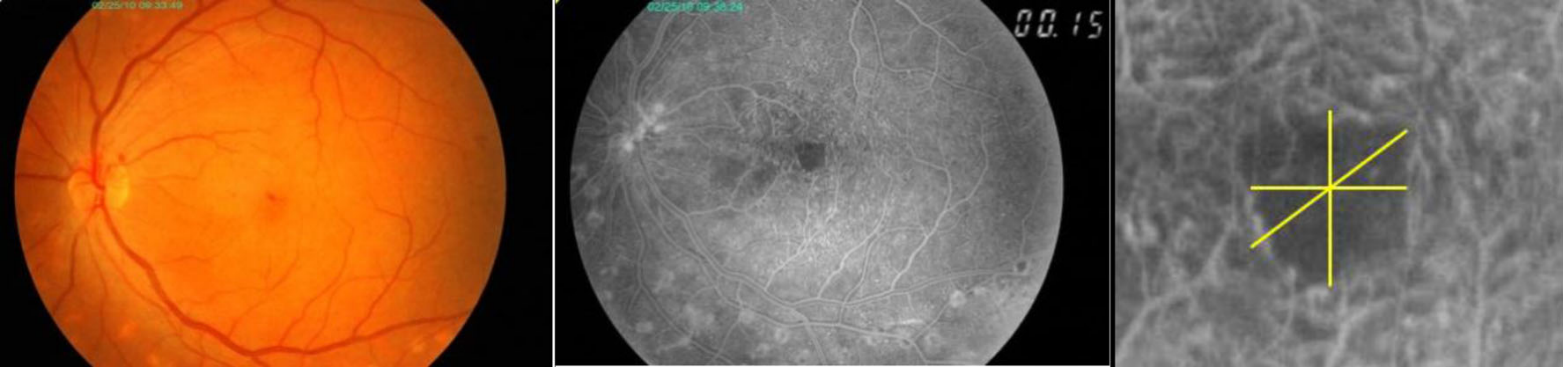
Sample color fundus photograph and early-phase angiogram. Magnified photo of FAZ presents measurements of horizontal, vertical, and largest linear dimensions

FAZ was manually outlined. The following dimensions were measured: horizontal and vertical linear dimensions passing through the presumed foveal center, as well as the greatest and smallest linear dimensions passing through the presumed foveal center in any meridian (Fig. 2). Then the arithmetic mean was calculated from the horizontal and vertical linear dimensions.

CSME was excluded based on clinical examination and presence of leakage on fluorescein angiograms. Patients were divided into two groups according to the severity of damage to the FAZ outline: ≤ grade 2 (*n* = 28) and ≥ grade 3 (*n* = 23).

**Fig. 3:**
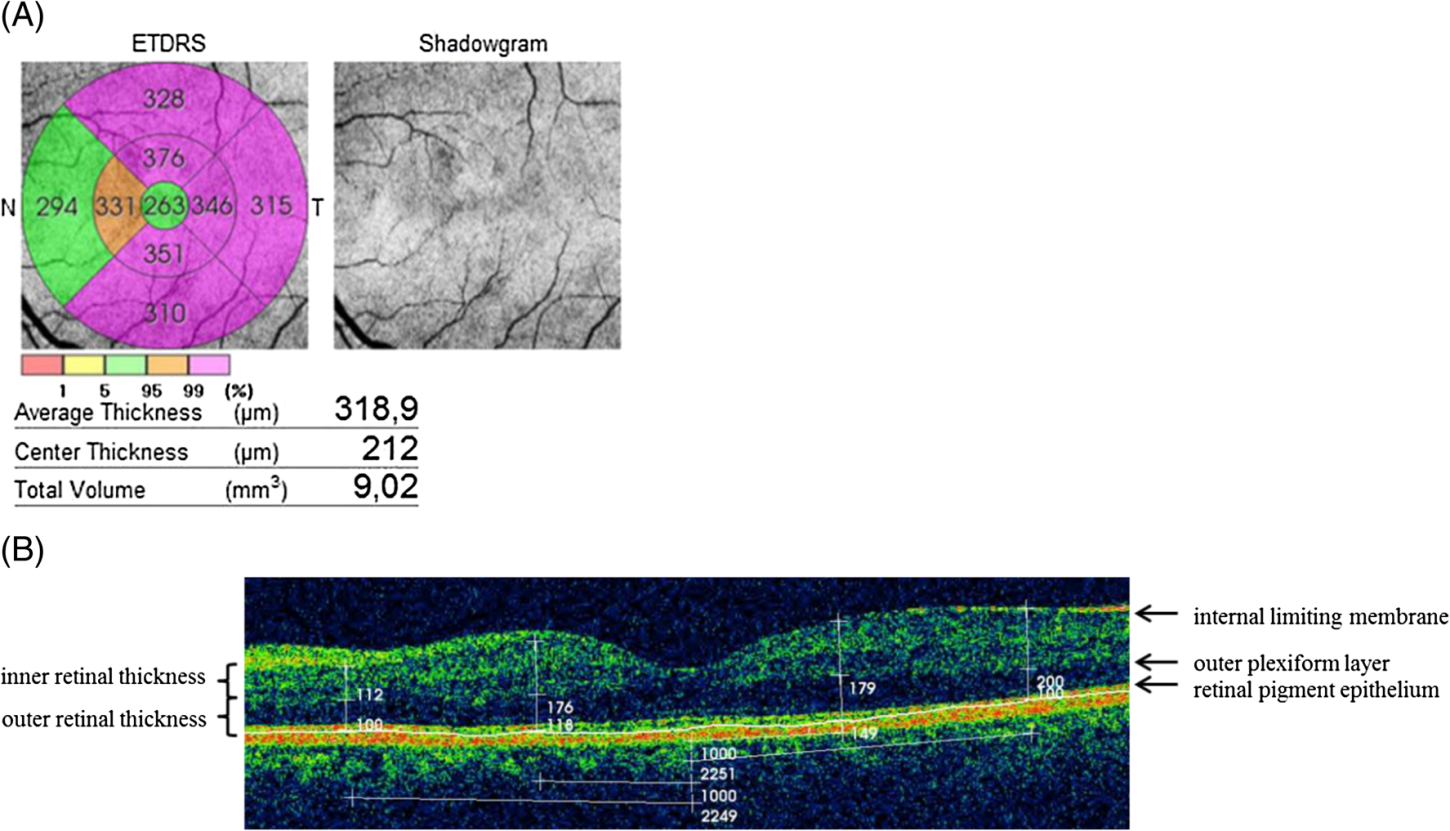
Sample OCT image. **a** The total volume and averaged thickness in the nine ETDRS fields were measured automatically: central macular (central field within a 500 μm radius), four parafoveal (within a 500–1500 μm radius) and four perifoveal subfields (within a 1500–3000 μm radius) [24]. **b** The outer and inner retinal thickness was measured manually on the horizontal scans in the middle of the parafoveal region (1000 μm from the fovea) and in the middle of the perifoveal region (2250 μm from the fovea) nasally and temporally. The outer thickness was defined as the distance between retinal pigment epithelium to and including the outer plexiform layer, while the inner thickness corresponded to the area from and including the inner nuclear layer and the inner retinal surface (temporally)/nerve fiber layer (nasally)

SD-OCT was performed using TOPCON 3D OCT-1000 (Topcon Medical Systems Inc., Oakland, CA, USA) with axial resolution of 6 μm. Retinal thickness, volume, structure, and vitreomacular interface were assessed. Retinal thickness, defined as the distance between the inner retinal surface and retinal pigment epithelium, was measured automatically. The averaged thickness in the nine ETDRS fields was analyzed [24]. Next, the outer (from the retinal pigment epithelium to and including the outer plexiform layer) and inner retinal thicknesses [from and including the inner nuclear layer to the inner retinal surface (temporally)/nerve fiber layer (nasally)] was measured manually as demonstrated in Fig. 3. Ganglion cell layer, defined as the distance between the inner plexiform layer and nerve fiber layer, was separately assessed.

Retinal structure evaluation employing a scan traversing horizontally through the presumed foveal center included continuity of the external limiting membrane and inner segment/outer segment junction (within the distance of 500 μm nasally and temporally from the fovea), identification of a boundary between the inner and outer retina, and identification of ganglion cell layer boundaries.

The following exclusion criteria were met: presence of CSME (based on slit-lamp biomicroscopy and FA), prior posterior segment surgery or intravitreal injections, macular laser photocoagulation, ametropia ≥3.0 diopters, macular changes resulting from other ocular diseases, and insufficient quality of fluorescein angiograms or OCT images. Moreover, patients with cilioretinal artery detected on fluorescein angiograms were excluded. Cilioretinal artery may be present in 20 % of normal eyes; its significance is derived from the fact that in such a case the inner layer of the macula is dually supplied by the choroidal and retinal circulations.

Statistical analyses were performed with the SPSS 16.0 (SPSS Inc., Chicago, IL, USA). Data were presented as means ± standard deviations or medians (min–max). Independent samples *t*-test for continuous data with normal distributions, Mann–Whitney U test for continuous data with non-normal distributions, and chi-square analysis for categorical variables were used to test the difference between the groups. Correlation was performed with Spearmann’s test. *P* value less than 0.05 was considered statistically significant.

## Results

Table 1 shows the clinical characteristics of 51 eyes of 51 patients with severe NPDR and PDR. None of the patients presented with CSME. Patients were divided into two groups according to their FAZ outline: ≤ grade 2 (*n* = 28) and ≥ grade 3 (*n* = 23).The patients in the second group were younger. There were no statistically significant differences in sex or diabetic retinopathy distribution between the groups.

Comparison of retinal thickness measurements and total volume values in relation to FAZ outline is presented in Table 2. In the case of FAZ outline ≤ grade 2, the retina was generally thicker and possessed a larger volume; however, the differences were not statistically significant. This tendency was not valid for the ganglion cell layer thickness.

**Table 2:** Comparisons of retinal volume and thickness in relation to FAZ outline

		All	FAZ outline grade 0, 1, 2	FAZ outline grade 3, 4	*P*
Total retinal thickness (μm)*	CMT	236.4 ± 30.1	241.0 ± 28.9	233.3 ± 31.9	0.449
	paraS	298.3 ± 32.5	303.5 ± 36.7	296.5 ± 28.9	0.453
	paraT	281.5 ± 36.1	291.0 ± 32.3	273.6 ± 41.6	0.179
	paraI	287.6 ± 31.7	292.3 ± 25.2	284.1 ± 37.2	0.467
	paraN	296.8 ± 28.2	300.5 ± 26.6	294.6 ± 32.4	0.526
	periS	272.1 ± 24.4	273.4 ± 25.1	270.6 ± 24.0	0.689
	periT	257.6 ± 30.8	263.3 ± 24.9	256.0 ± 40.2	0.189
	periI	264.1 ± 23.7	261.8 ± 20.0	266.7 ± 26.6	0.465
	periN	284.7 ± 25.7	283.6 ± 20.5	286.2 ± 31.3	0.734
Outer retinal thickness (μm)**	periT	112.9 ± 21.1	116.5 ± 19.2	107.2 ± 24.0	0.138
	paraT	126.6 ± 21.7	129.0 ± 23.7	121.6 ± 20.3	0.306
	paraN	117.7 ± 20.5	115.6 ± 20.6	119.6 ± 21.8	0.398
	periN	103.0 ± 15.4	99.0 ± 12.1	110.1 ± 19.5	0.011
Inner retinal thickness (μm)**	periT	142.4 ± 34.2	146.0 ± 31.8	140.3 ± 43.1	0.511
	paraT	157.6 ± 25.6	161.9 ± 27.9	154.9 ± 25.7	0.278
	paraN	159.6 ± 30.6	169.1 ± 29.6	146.7 ± 27.7	0.011
	periN	130.3 ± 18.1	132.8 ± 18.4	127.1 ± 17.5	0.291
Ganglion cell layer thickness (μm)***	periT	83.7 ± 14.7	81.0 ± 15.9	90.1 ± 9.0	0.202
	paraT	89.2 ± 12.6	92.1 ± 11.2	84.0 ± 13.8	0.132
	paraN	87.1 ± 15.5	86.5 ± 15.6	88.9 ± 16.1	0.839
	periN	69.1 ± 13.2	65.3 ± 10.4	76.3 ± 17.4	0.078
Total volume (mm^3^)*		7.74 ± 0.64	7.80 ± 0.61	7.72 ± 0.77	0.599

FAZ = foveal avascular zone; CMT = central macular thickness; peri = perifoveal; para = parafoveal; S = superior; T = temporal; I = inferior; N = nasal; *n = 51; **n = 47; ***n = 28

The following median (min–max) FAZ dimensions were obtained: horizontal 1160 μm (532–6539), vertical 976 μm (391–4366), mean 1102 μm (461–5452); greatest 1261 μm (537–6539) and smallest 867 μm (360–2674). The FAZ dimensions were statistically significantly greater in the group with more destroyed FAZ outline (*p* < 0.001).

Correlation of retinal thickness and volume with FAZ size proved not significant (Table 3).

**Table 3:** Correlation of the retinal volume and thickness in relation to FAZ linear dimensions. *P* values are given in the table

Linear dimensions (μm)		Horizontal	Vertical	Greatest	Smallest	Mean
Total retinal thickness (μm)	CMT	0.346	0.476	0.391	0.528	0.381
	paraS	0.903	0.781	0.803	0.767	0.913
	paraT	0.254	0.182	0.320	0.249	0.215
	paraI	0.938	0.758	0.780	0.553	0.867
	paraN	0.736	0.705	0.744	0.698	0.883
	periS	0.583	0.806	0.951	0.618	0.702
	periT	0.217	0.261	0.288	0.258	0.228
	periI	0.922	0.630	0.469	0.944	0.761
	periN	0.542	0.925	0.456	0.835	0.728
Outer retinal thickness (μm)	periT	0.862	0.572	0.903	0.653	0.825
	paraT	0.659	0.416	0.578	0.665	0.430
	paraN	0.684	0.321	0.550	0.258	0.497
	periN	0.240	0.457	0.230	0.487	0.224
Inner retinal thickness (μm)	periT	0.926	0.885	0.797	0.767	0.974
	paraT	0.897	0.693	0.866	0.479	0.904
	paraN	0.890	0.480	0.851	0.802	0.673
	periN	0.409	0.410	0.449	0.317	0.342
Ganglion cell layer thickness (μm)	periT	0.491	0.078	0.171	0.100	0.175
	paraT	0.312	0.682	0.625	0.764	0.428
	paraN	0.854	0.508	0.774	0.608	0.894
	periN	0.240	0.037	0.101	0.082	0.115
Total volume (mm^3^)		0.914	0.882	0.766	0.723	0.867

CMT = central macular thickness; peri = perifoveal; para = parafoveal; S = superior; T = temporal; I = inferior; N = nasal

The comparison of the retinal structure between group 1 (FAZ outline ≤ grade 2) and group 2 (FAZ outline ≥ grade 3) did not reveal any significant differences. The assessed features included: continuity of the external limiting membrane (*p* = 0.769), and inner segment/outer segment junction (*p* = 0.120). Identification of the boundary between the inner and outer retina was not possible in one out of 28 patients in group 1 (FAZ outline ≤ grade 2) and three out of 23 subjects in group 2 (FAZ outline ≥ grade 3) (*p* = 0.211). The identification of ganglion cell layer boundaries was not possible in 29 % of patients in group 1 (FAZ outline ≤ grade 2) and 65 % of subjects in group 2 (FAZ outline ≥ grade 3) (*p* = 0.009).

## Discussion

The study has demonstrated that, at least at present with described methods, in patients with severe NPDR and PDR without CSME:

1. It is not possible to predict the FAZ outline/size based solely on the measurement of thickness and retinal structure evaluation in OCT.
2. There is no significant association between FAZ outline and: retinal volume, total thickness, and thickness of the outer and inner retina and ganglion cell layer.
3. There is no significant association between FAZ outline and the following retinal structural characteristics: continuity of the external limiting membrane and inner segment/outer segment junction, and identification of the inner/outer retina boundary.
4. Identification of ganglion cell layer boundaries was more difficult in more advanced FAZ outline grades.
5. FAZ size is not correlated with the retinal thickness and volume.
6. FAZ size is significantly larger in more advanced FAZ outline grades.

We did not find significant thickness differences, and consequently volume, in association with the severity of FAZ damage. The ischemic changes could theoretically lead to atrophy and result in reduced thickness. On the other hand, these changes might be compensated by edematous enlargement of glial cells, as explained by Yanoff et al. and Fine et al. [25, 26]. Another explanation might be that most of the cells in the area of perifoveal hypo- and nonperfusion survive, though in a semi-starved state. This hypothesis cannot be proved based on the currently available OCT images, as their resolution does not allow us to count individual cells. The same mechanisms could account for the lack of difference in the inner retinal thickness in relation to FAZ damage. The findings of Sanchez-Tocino et al. and Lattanzio et al. [11, 12] support our hypothesis. They showed that there were no significant differences in average thickness in any area between NPDR and PDR without CSME. Similarly, Ozdek et al., who evaluated patients with diabetic ischemic maculopathy as well as with CSME, did not find a significant difference in foveal thickness between the eyes with NPDR and PDR and in eyes with and without macular ischemia [17]. Furthermore, it is known that FA evaluates the superficial capillary plexus. In cases where superficial capillary dropout is present, deep capillary plexus still may be intact and vice versa. Although FAZ outline was not destroyed (grade ≤ 1) in 14 (27.5 %) of our patients, we found evidence of ischemic changes within the retina as reflected by severe NPDR and PDR. Diabetes causes reduced choroidal circulation [27, 28] and thickness [2–4], which might lead to hypoxia of the outer retina. We did not assess the choroidal thickness and flow, but based on these reports such changes may be assumed to be present, especially in patients with advanced DR stages. Nevertheless, we did not find a difference in the outer retinal thickness in association with FAZ outline. This could be attributed to the semi-starved state or the compensation of decreased number of photoreceptors by the swelling of the remaining cells. However, it seems more probable that even in advanced stages of DR, choroidal blood supply is sufficient. It is worth noting that approximately 70 % of the total ocular blood flow can be found in the choriocapillaris with its fenestrated endothelium. Choroid is characterized by high flow and low resistance [29, 30]; thus, with its reserve even substantial reduction of choroidal circulation should not affect blood supply to the outer retina.

Blood supply to the ganglion cell layer is derived from the superficial capillary plexus. Damage of this structure, reflected by more advanced FAZ outline grades, should contribute to changes within this layer. Byeon et al. suggested that changes in the thickness of the foveal ganglion cell layer represent alterations in foveal circulation. Consequently, evidence of the damage to the ganglion cell layer on OCT may be a good indicator of macular ischemic damage in eyes with DR [19]. Van Dijk et al. also reported decreased retinal ganglion cell layer thickness in patients with type 1 diabetes [20]. We were intrigued by this idea, and decided to investigate it in our study. Unfortunately, in almost half of the patients with advanced DR stages, particularly those with more destroyed FAZ outline, we were not able to distinguish the ganglion cell layer boundaries. Furthermore, our results did not confirm the previously reported findings, as no association between FAZ outline and ganglion cell layer thickness was found, most likely due to the low number of patients in whom we were able to identify the borders and measure the thickness.

We have not found an association between FAZ outline and retinal structural features such as: continuity of the external limiting membrane and inner segment/outer segment junction (within 500 μm nasally and temporally from the fovea), and identification of a boundaries of the ganglion cell layer and between inner and outer retina. The latter was possible in almost every patient. It might be attributed to the dual circulation to the retina and the presence of the watershed zone at the outer plexiform layer. As suggested earlier, choroidal circulation supplying outer retina appeared to be sufficient in our patients, even in the face of reduced blood supply to the inner retina. Furthermore, continuity of the external limiting membrane and inner segment/outer segment junction, belonging to the outer retina, seems not to differ in association with FAZ outline damage for the same reason. In contrast, the identification of ganglion cell layer boundaries was significantly more difficult in patients with more severe FAZ outline damage (impossible in 29 % in ≤ grade 2 versus 65 % in ≥ grade 3, *p* = 0.009). As the ganglion cell layer is supplied by the superficial capillary plexus, the damage of which is reflected in FA by FAZ outline destruction, the association appears to be direct. Yeung et al. showed that loss of retinal layers, more prominent within the inner retina, corresponded to areas of capillary non-perfusion on FA [18]; our results support this finding. In conclusion, based on our results, we believe that the loss of ganglion cell layer boundaries, and not necessarily the thickness, is associated with the progression of FAZ outline damage.

FAZ size and, in particular, outline constitute indicators of inner retina perfusion within the macula. The ischemic state of the macula corresponds to the destruction of the FAZ outline and related enlargement [5, 8, 24]. Our study confirmed the association between FAZ size and outline. Also, our results indicate that FAZ size does not correspond to retinal thickness or volume. This conclusion seems reasonable, as FAZ size is related to FAZ outline; the latter is not associated with retinal thickness or volume. Moreover, even in healthy subjects FAZ size varies widely [1]. Furthermore, Byeon et al. suggested that FAZ size does not represent the degree of damage to the inner retina [19].

Weaknesses are present within our study, including the retrospective character of research and limited number of patients. In order to select a particular group of patients (severe NPDR and PDR) and to meet the exclusion criteria, in particular the absence of CSME, fluorescein angiograms of 315 consecutive patients (almost 600 eyes) with any stage of DR were reviewed. However, only 51 eyes (8.5 %) were suitable for further evaluation.

The following points highlight the strengths of our investigation with regard to study design and material and methods:

1. Association between retinal thickness/volume and FAZ outline;
2. Focus on a particular group of patients (presenting with severe NPDR and PDR, without CSME), who are often excluded from other studies;
3. Subdivision of the retinal thickness in the inner and outer retina to investigate the influence of both blood supplies;
4. Measurement of ganglion cell layer thickness and assessment of its boundaries.

In the literature, contradictory data is presented on the role of the two imaging methods, OCT and FA, in the detection of ischemic diabetic maculopathy [16–19]. Our study did not provide a definitive answer to this issue. Nevertheless, at the present time it seems that OCT cannot replace AF in this regard. FA enables the evaluation of foveal perfusion, which, according to our study, could not be predicted based on OCT.
